# Supplementary material for: Long-term voluntary exercise reveals limited translation of hippocampal molecular responses into neuroprotection in 5xFAD mice
Source: bioRxiv. 2026 Sep 22:2026.09.16.752181. Preprint. [Version 1] doi: 10.64898/2026.09.16.752181 (PMC13621997; doi:10.64898/2026.09.16.752181)

Supplementary Figure 1

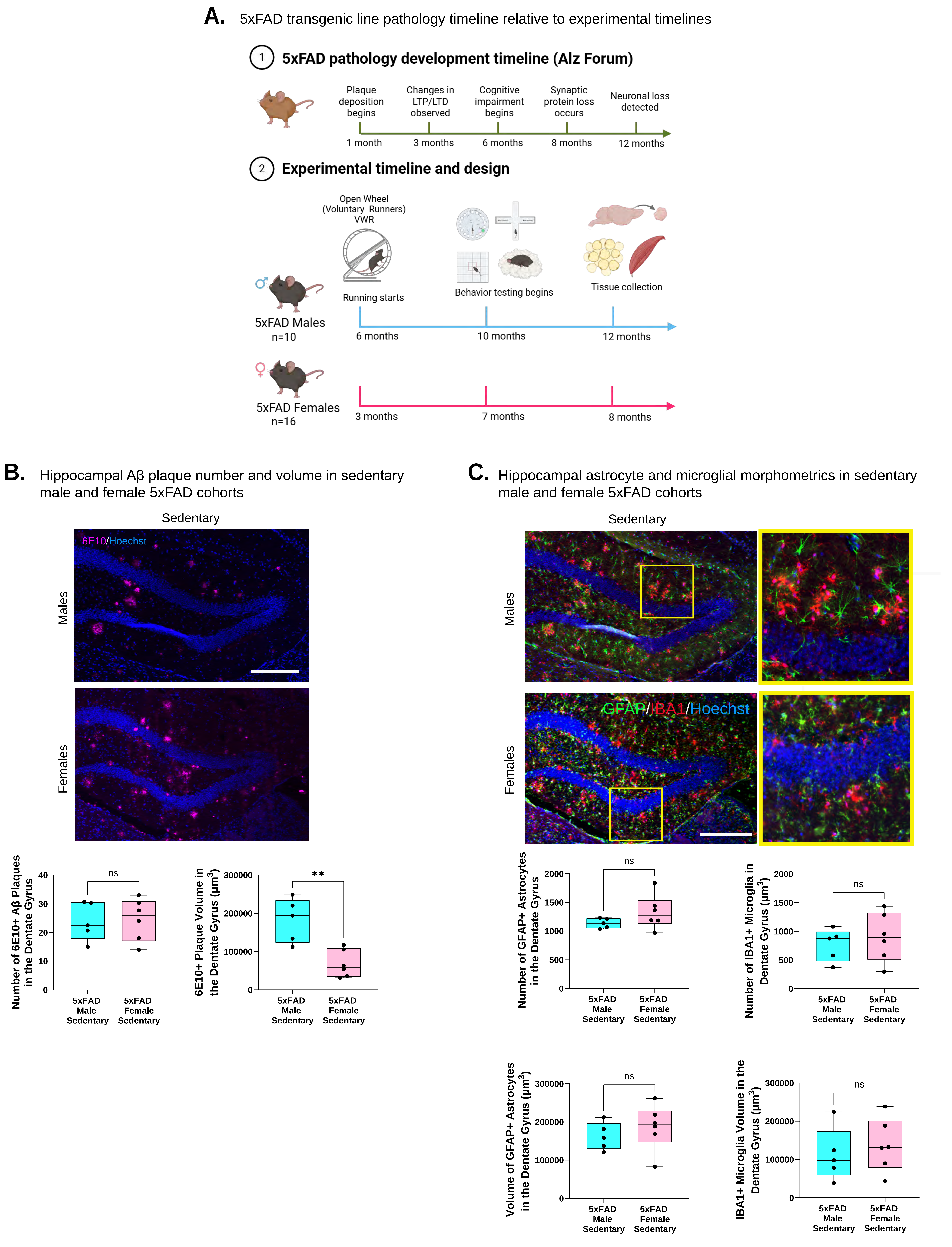

Supplementary Figure 2 (males)

A. Principal component analysis of male 5xFAD hippocampal transcriptomes

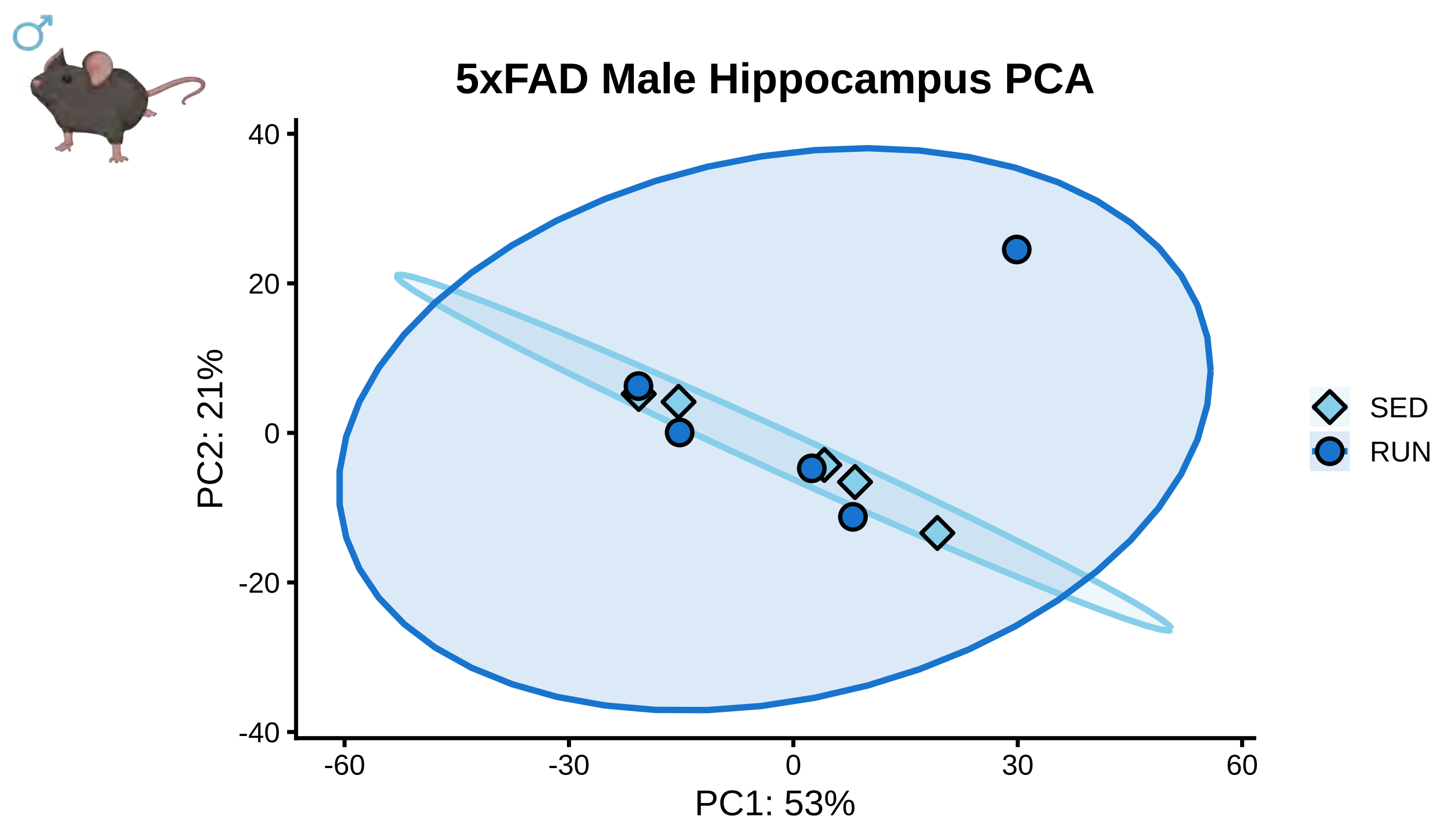

B. Volcano plots for differential gene expression of the male 5xFAD hippocampus

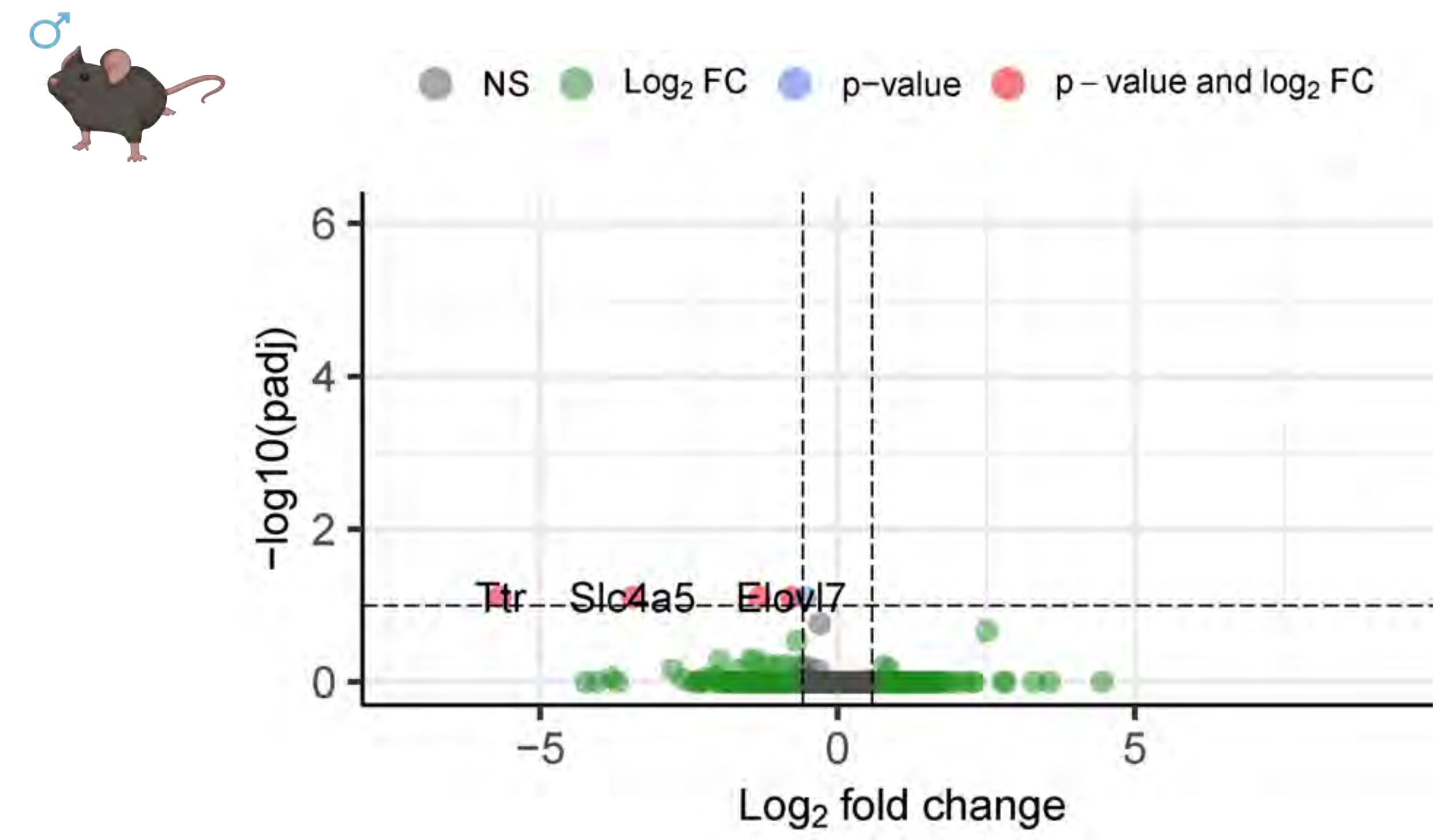

C. Top 30 GO Cell Compartment terms in runner male 5xFAD hippocampus

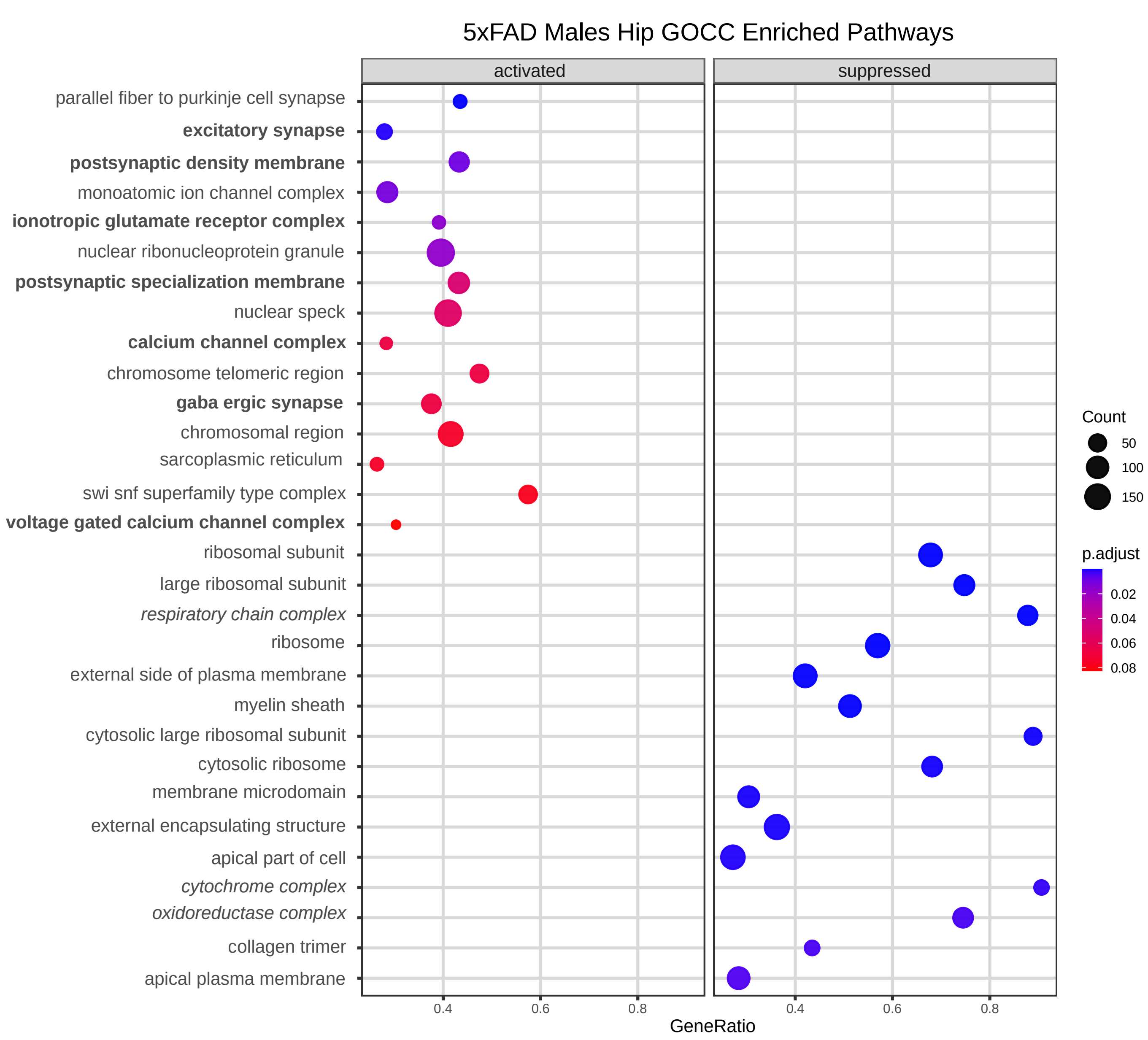

D. Top 30 GO Molecular Function terms in runner male 5xFAD hippocampus

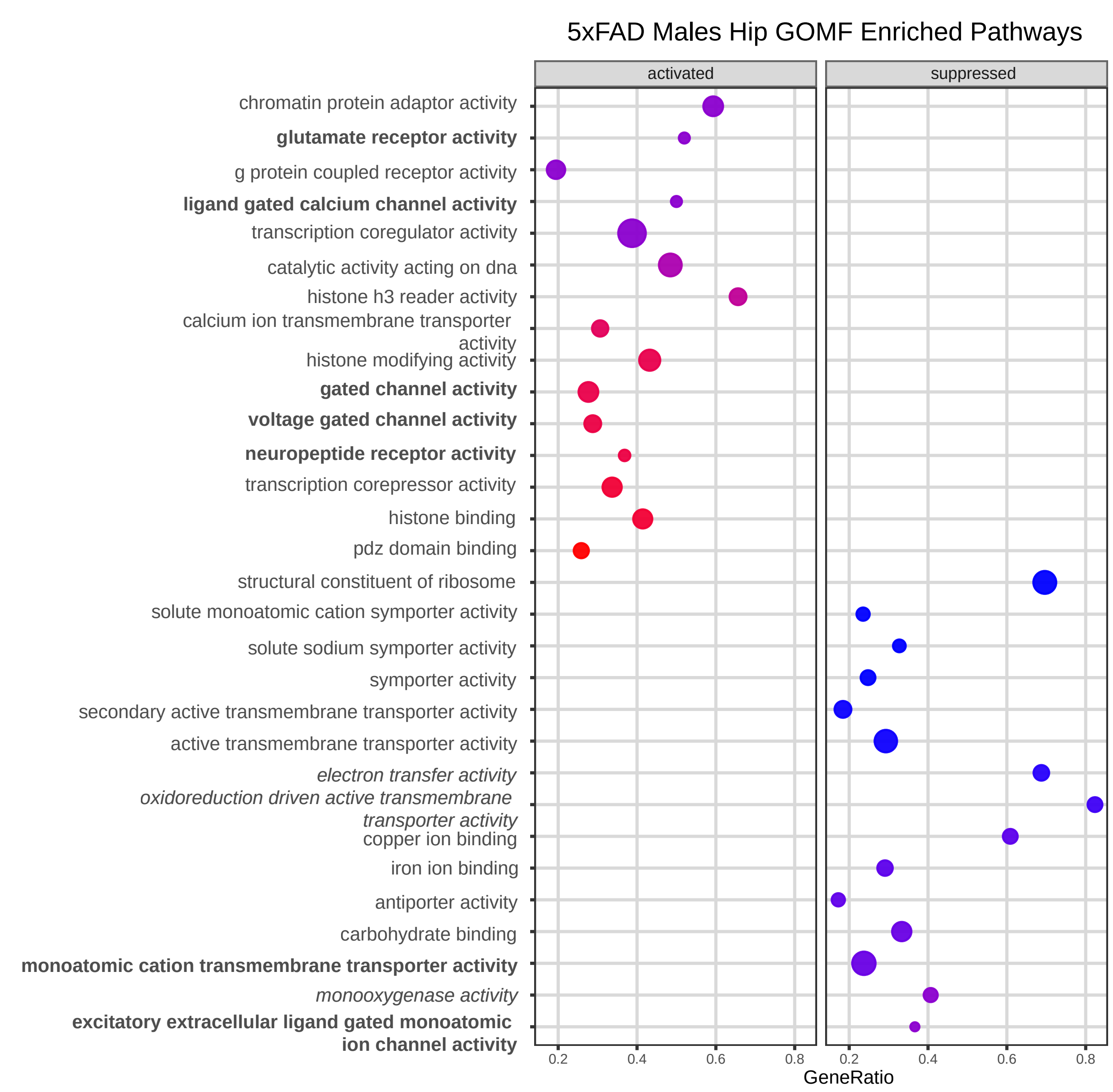

E. Quantification of average hippocampal astrocyte number

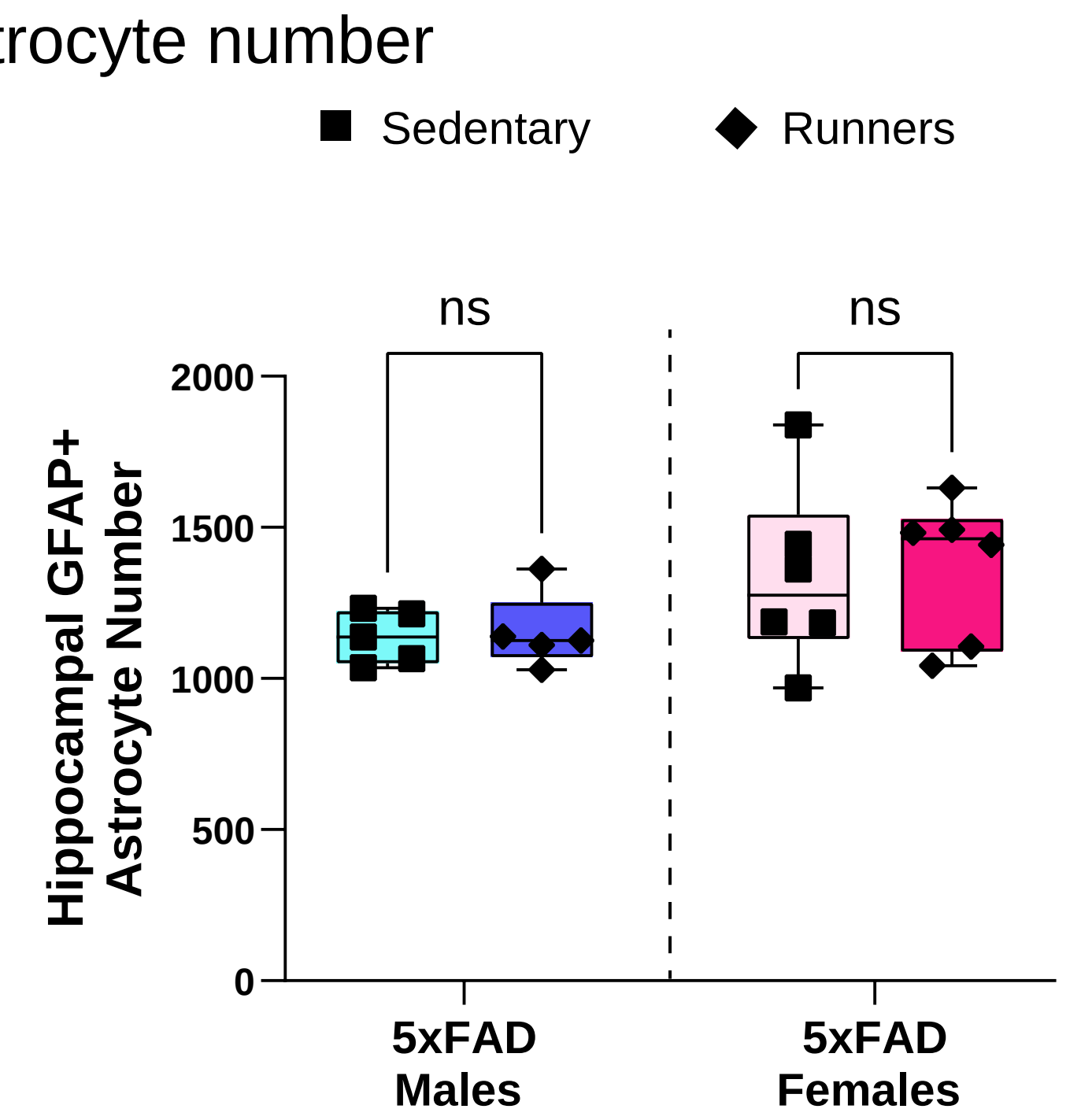

F. Size distribution of GFAP+ structures in the dentate gyrus of 5xFAD mice

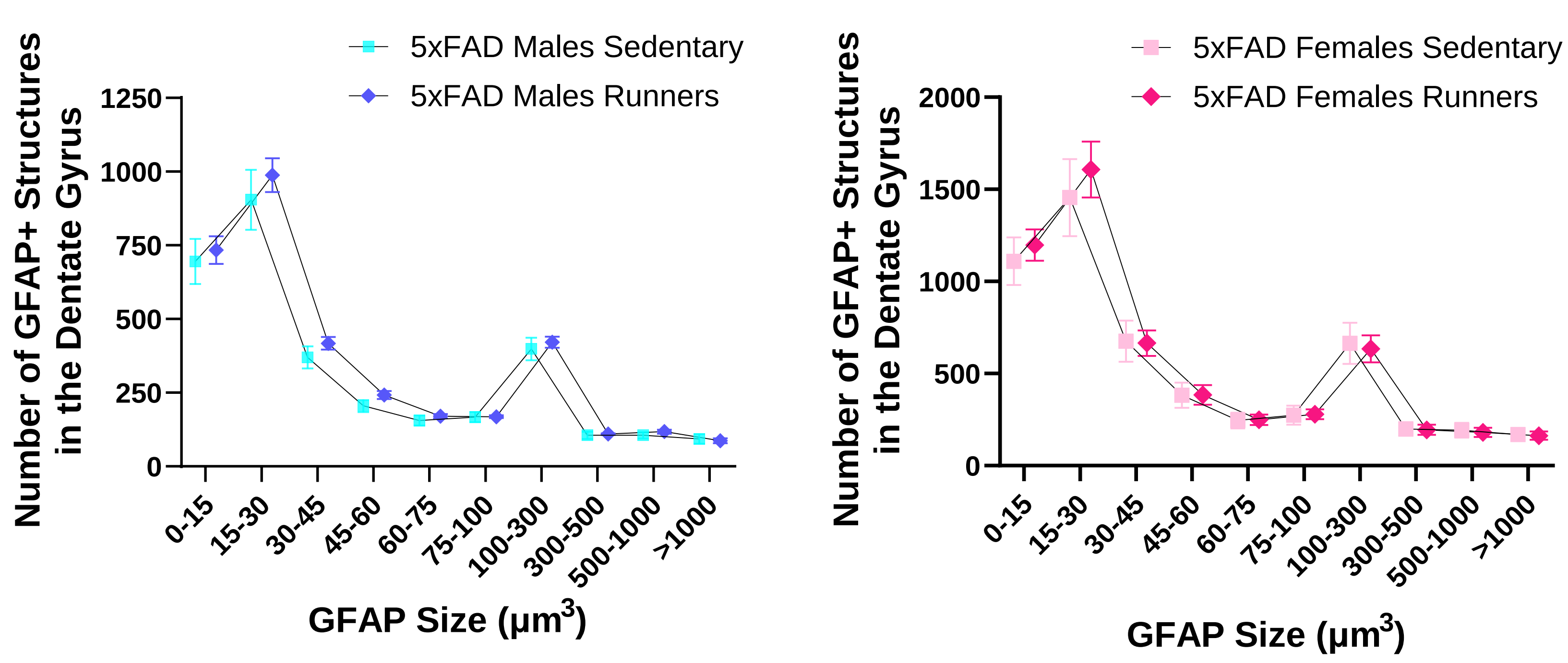

G. Representative images of microglial morphometric states in the 5xFAD dentate gyrus

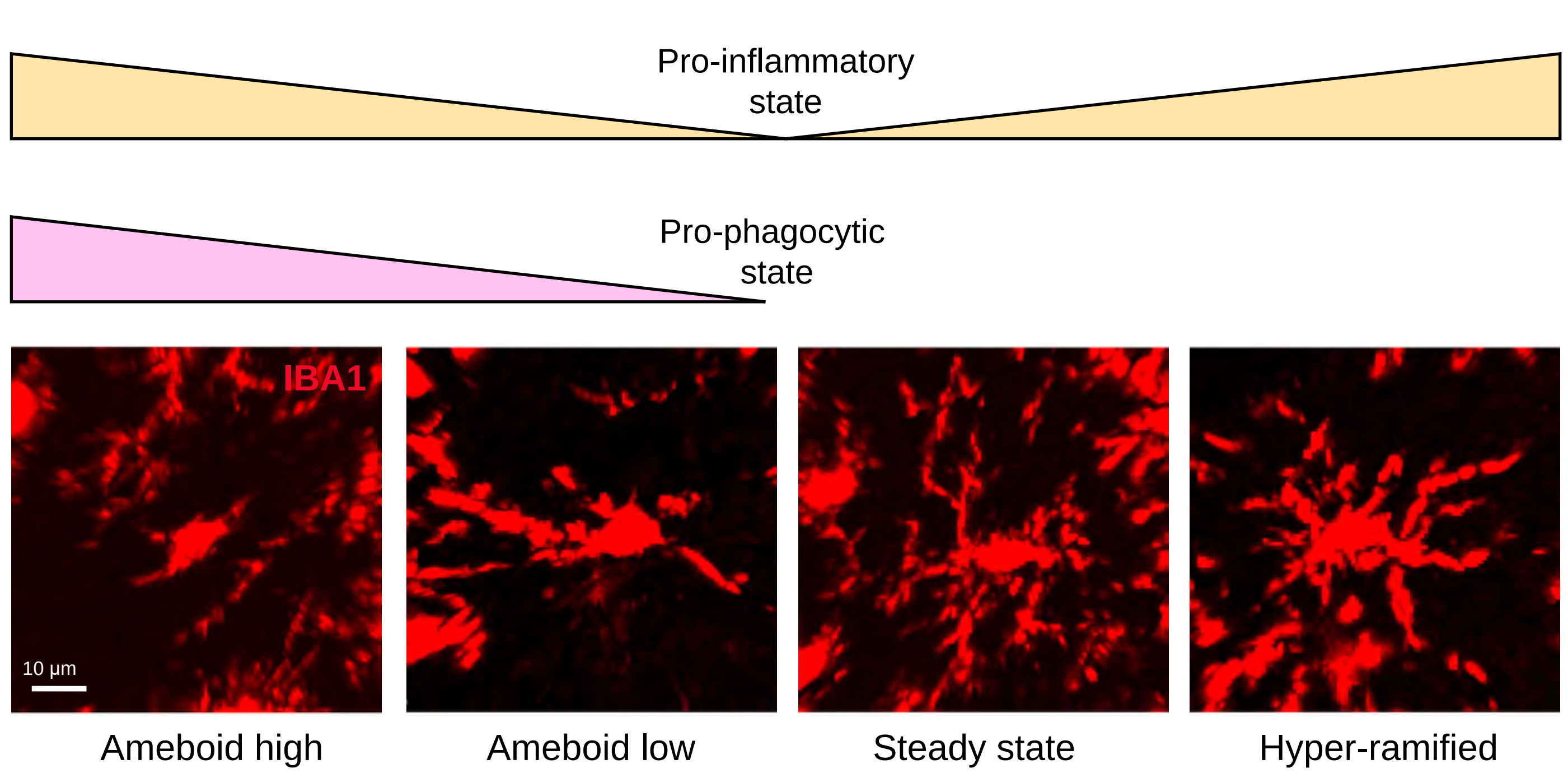

H. Quantification of IBA+ microglia shape

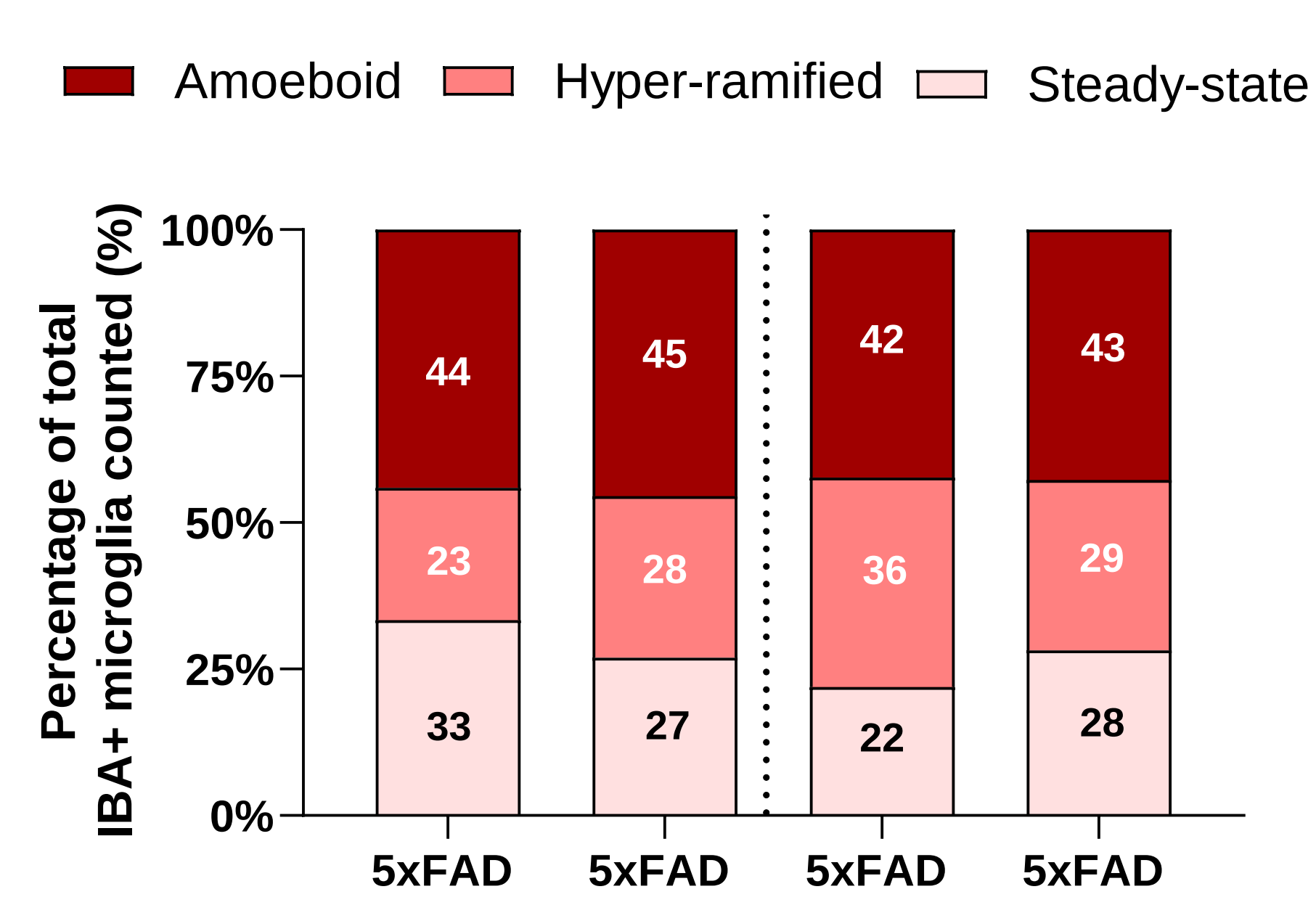

Supplementary Figure 3 (females)

A. Principal component analysis of female 5xFAD hippocampal transcriptomes

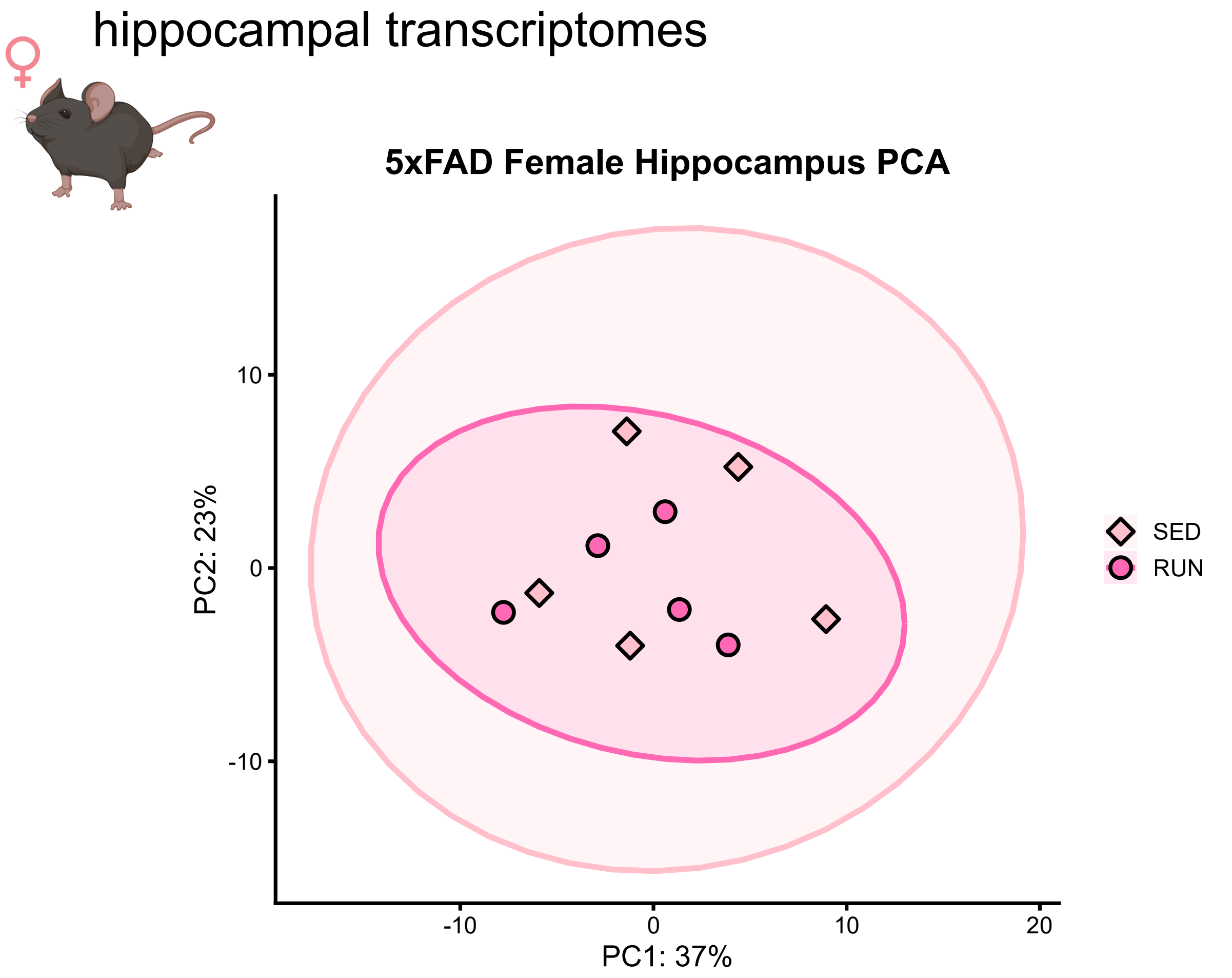

B. Volcano plots for differential gene expression of the female 5xFAD hippocampus

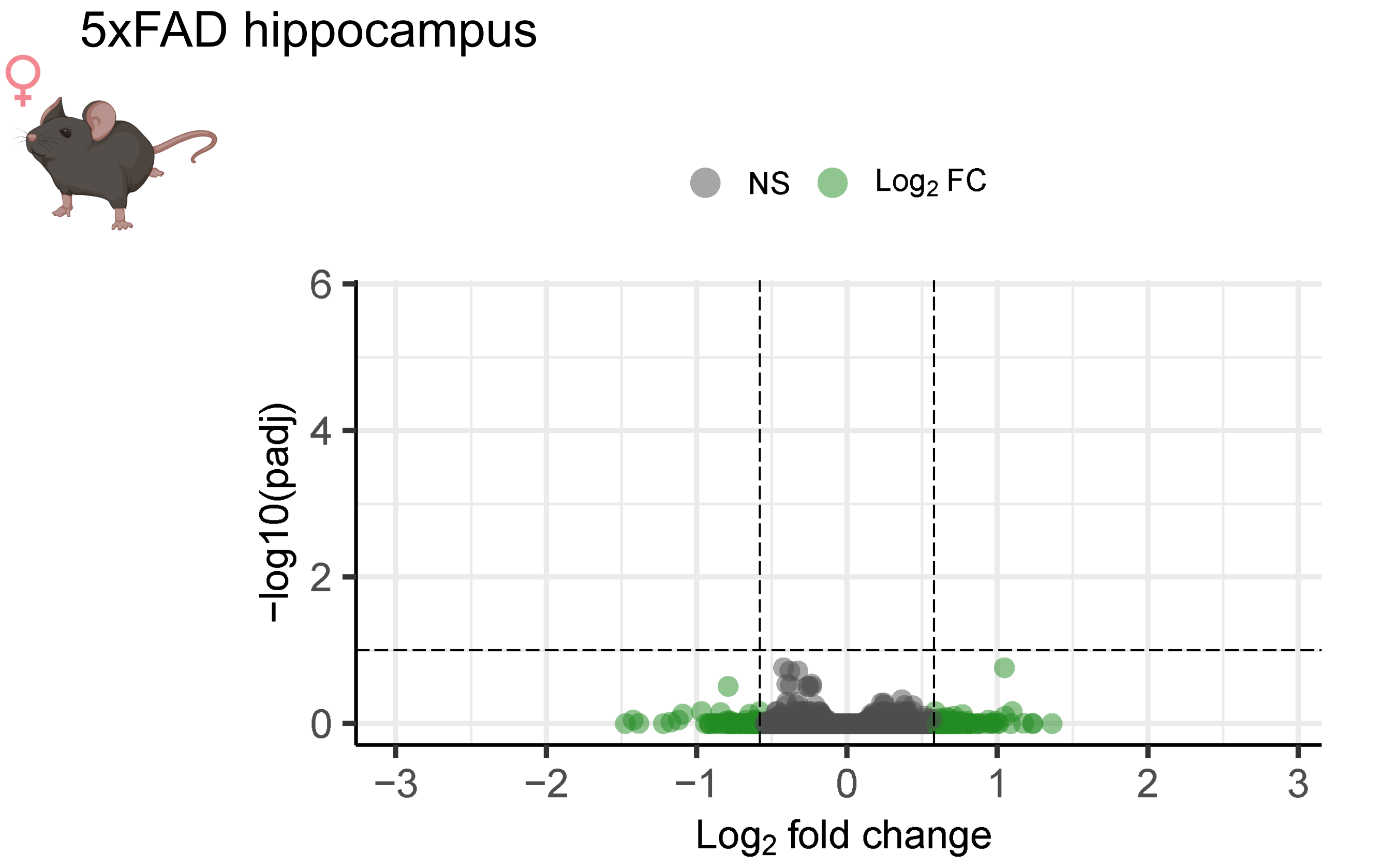

C. Top 30 GO Cell Component terms in runner female 5xFAD hippocampus

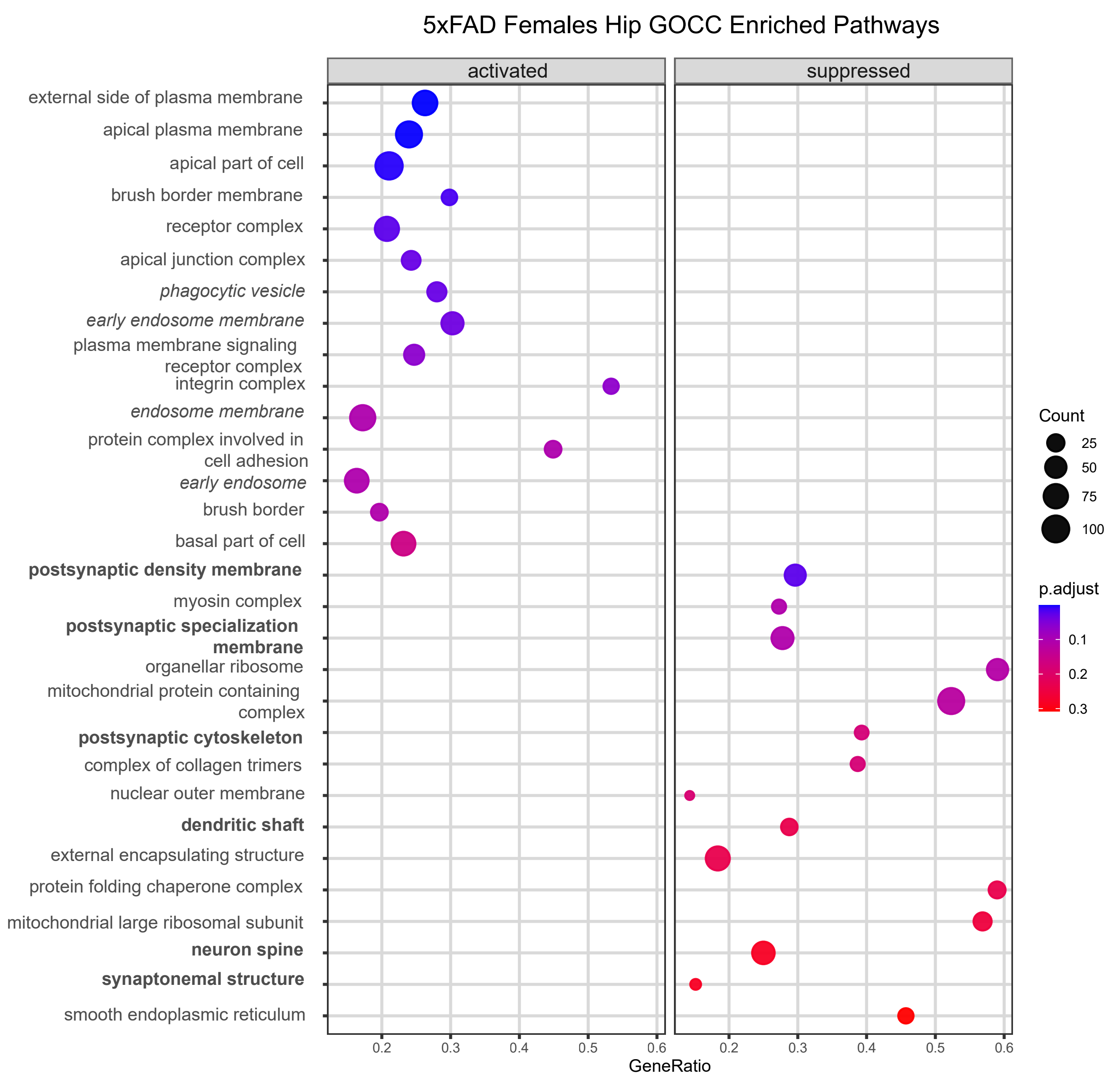

D. Top 30 GO Molecular Function terms in runner female 5xFAD hippocampus

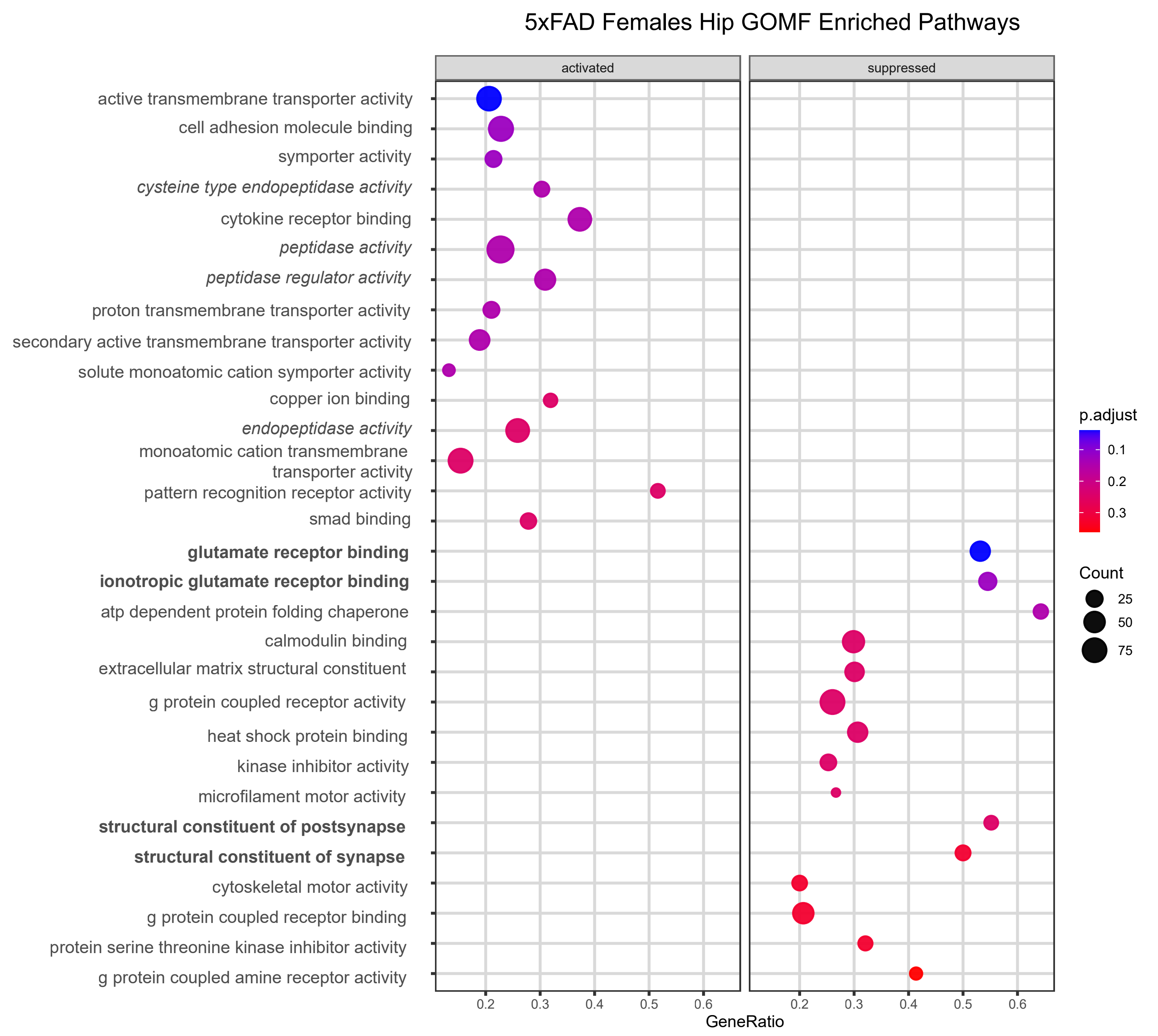

E. Gene expression pattern for selected Go Biological Process

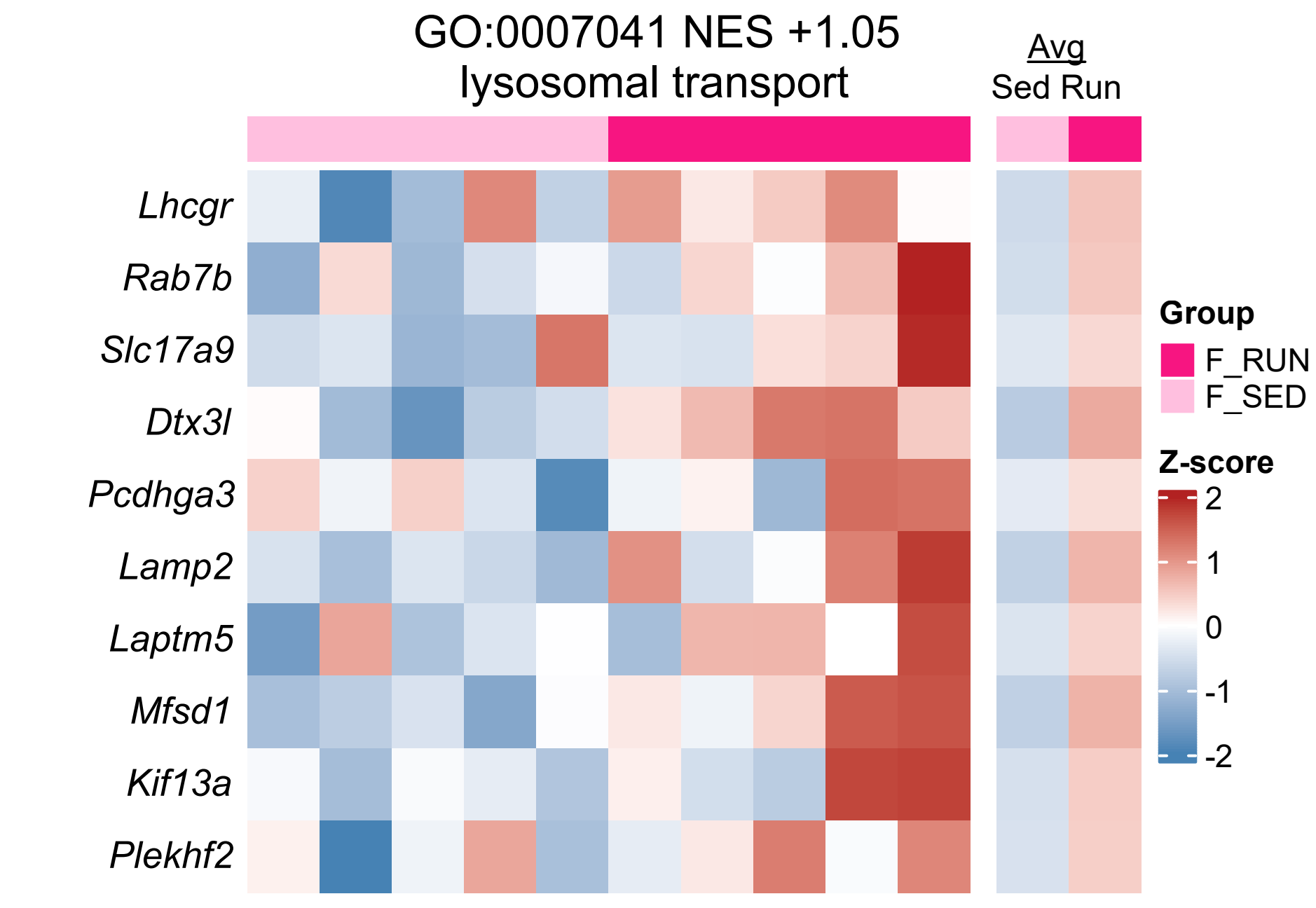

F. Quantification of hippocampal plaque number

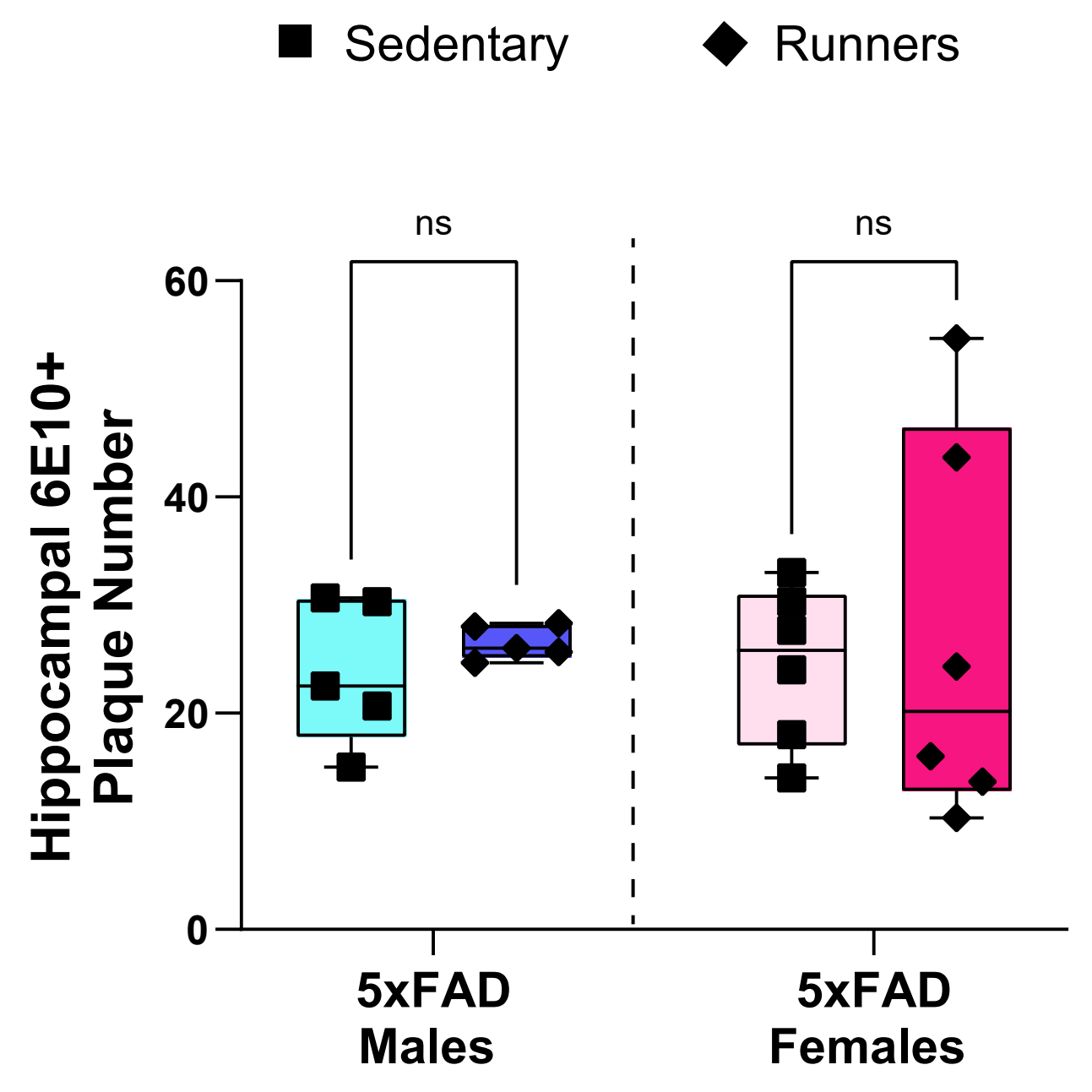

G. Quantification of hippocampal plaque size

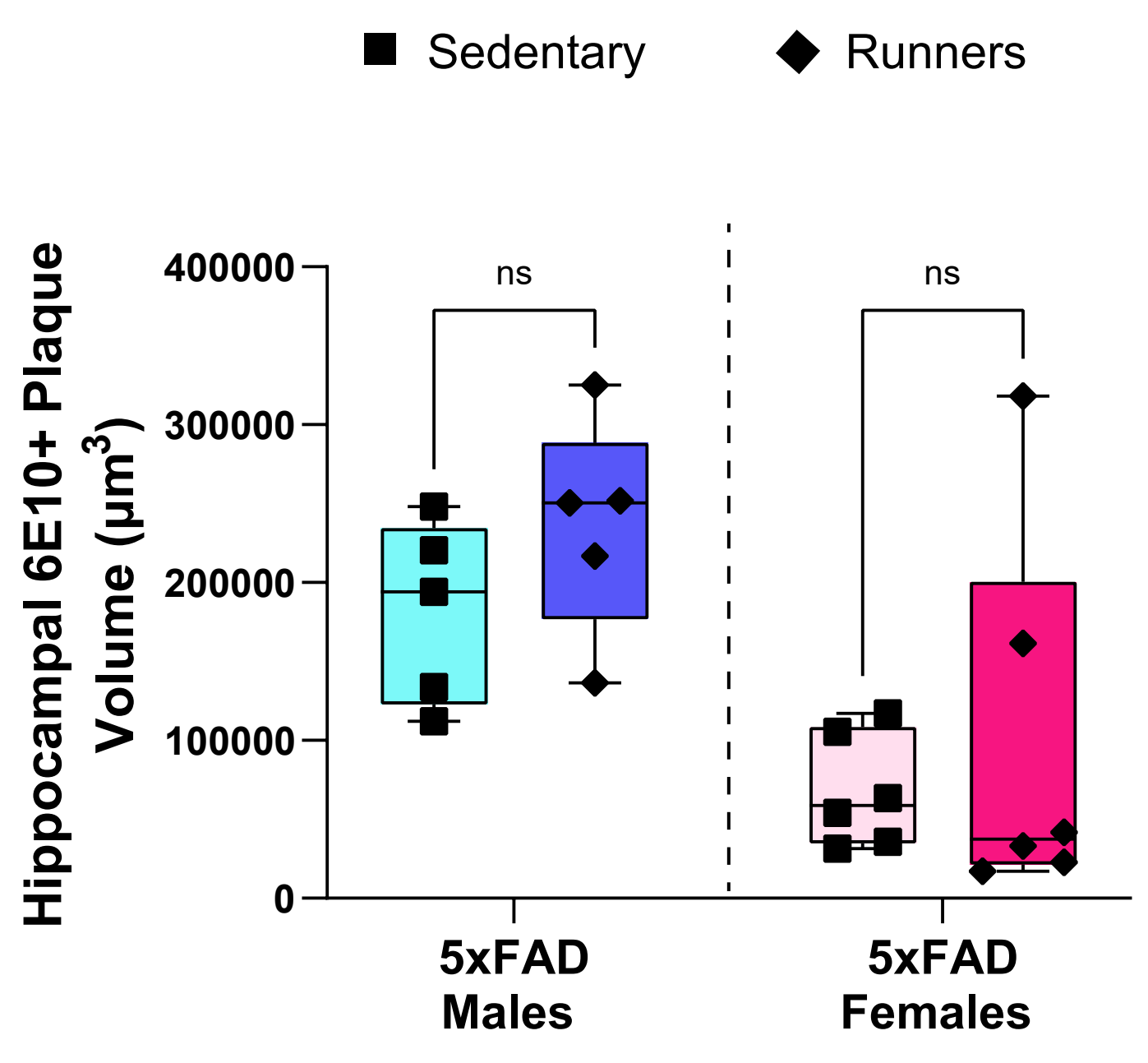

Supplementary Figure 4 (Behavior)

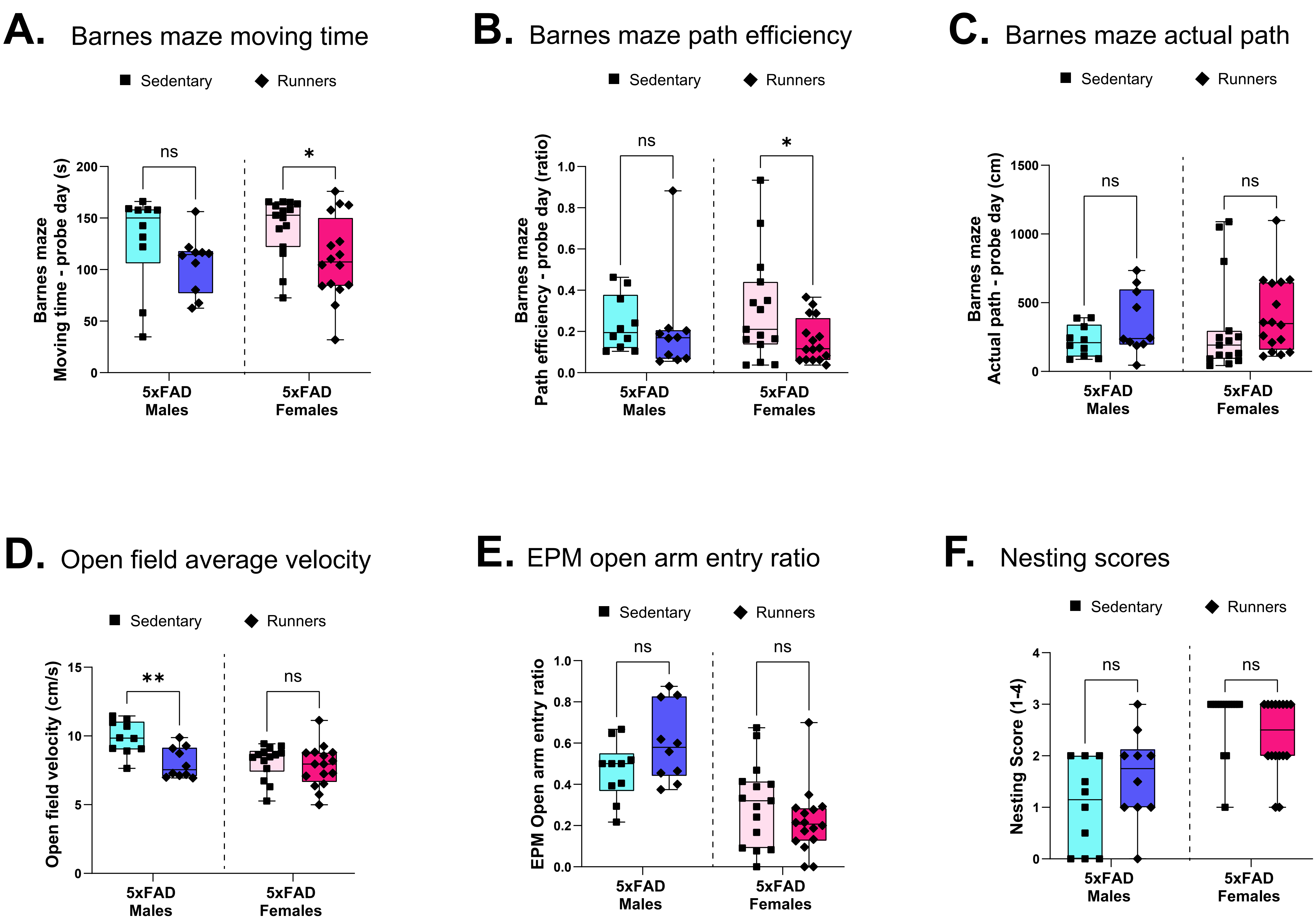

Supplementary Figure 5 (iWAT)

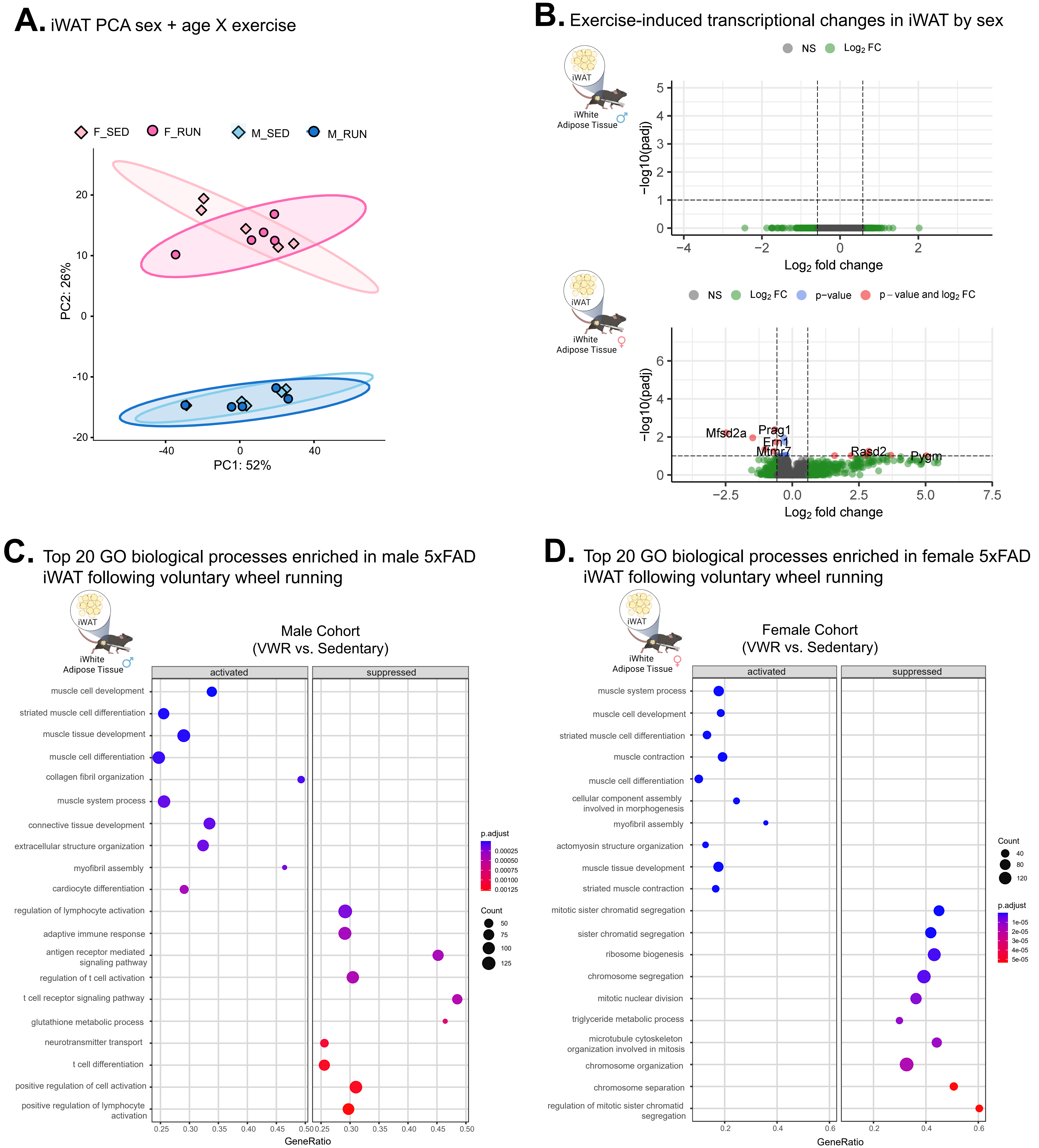

Supplement: Supplementary file 1 [file media-1.pdf]
